# Darapladib Ameliorates Radiation-Induced Skin Injury and Fibrosis by Lipoprotein-Associated Phospholipase A2 Inhibition

**DOI:** 10.3390/biom16091292

**Published:** 2026-09-07

**Authors:** Ji-Eun Park, Narae Kim, So-Ra Kim, Soo-Ho Lee, Yoon-Jin Lee, Kwang Seok Kim

**Affiliations:** 1 Divisions of Radiation Biomedical Research, Korea Institute of Radiological and Medical Sciences, Seoul 01812, Republic of Korea; rr105014@kirams.re.kr (J.-E.P.); narae0940@kirams.re.kr (N.K.); charming-ssol@hanmail.net (S.-R.K.); skarn88@kirams.re.kr (S.-H.L.); yjlee8@kirams.re.kr (Y.-J.L.); 2 School of Radiological and Medical Sciences, University of Science and Technology (UST), 75 Nowon-ro, Nowon-gu, Seoul 01812, Republic of Korea

**Keywords:** radiation-induced skin injury, skin fibrosis, Lp-PLA_2_, darapladib, regeneration

## Abstract

Current studies have elucidated the mechanisms of radiation-induced skin injury (RISI) and identified several medical countermeasures to reduce its severity. However, no treatment has yet proven effective in preventing or reversing radiation-induced skin fibrosis. Here, we show that radiation upregulates lipoprotein-associated phospholipase A2 (Lp-PLA_2_) expression and induces endothelial cell dysfunction, characterized by an increased DNA damage response and reduced tube-forming capacity and mitochondrial function. To define the role of Lp-PLA_2_ in the progression of RISI, we treated human dermal microvascular endothelial cells and the skin of SKH1 hairless mice with darapladib, a selective Lp-PLA_2_ inhibitor. In endothelial cells, darapladib attenuated radiation-induced cellular damage and suppressed endothelial-to-mesenchymal transition (EndoMT). In irradiated mouse skin, darapladib reduced radiation-induced inflammation, adipose tissue disruption, dermal thickness, and skin fibrosis. Notably, darapladib modulated macrophage polarization and inhibited radiation-induced macrophage infiltration in irradiated skin. These findings suggest that Lp-PLA_2_ inhibition may reveal potential targets for the treatment of RISI and other fibrotic skin diseases.

## 1. Introduction

Although radiation therapy (RT) is widely used to treat cancer, it also damages normal tissues, leading to acute and chronic side effects. In particular, soft tissues such as the skin and blood vessels frequently develop fibrosis after RT. Fibrosis is driven by a sustained, dysregulated wound-healing response following severe tissue injury, characterized by the accumulation of myofibroblasts [1]. Myofibroblasts are fibroblast-like cells that produce excessive extracellular matrix (ECM) proteins, including type I collagen and alpha-smooth muscle actin (α-SMA), resulting in abnormal connective tissue deposition and organ dysfunction [2]. Endothelial cells contribute to fibrosis by serving as a source of myofibroblasts through endothelial-to-mesenchymal transition (EndoMT), accompanied by microvascular rarefaction [3]. These processes are regulated by cytokines and growth factors such as tumor necrosis factor-alpha (TNF-α), interleukin-1beta (IL-1β), platelet-derived growth factor (PDGF), fibroblast growth factor-beta (FGF-β), and transforming growth factor-beta (TGF-β) [4], which promote the recruitment of immune cells. In particular, TGF-β is involved not only in normal processes such as epidermal maturation but also in the initiation and progression of fibrosis. Excessive TGF-β expression has been observed both in vitro and in situ in the dermis and epidermis of radiation-induced fibrotic lesions [5]. To treat fibrosis, various approaches targeting cytokines, chemokines, matrix metalloproteases (MMPs), angiogenic factors, and antioxidants have been proposed in an effort to inhibit TGF-β and its downstream signaling pathways, which play a central role in fibrogenesis [6]. These findings underscore the importance of establishing robust models of radiation-induced fibrosis to facilitate the development of effective therapies. However, there is still no clinically effective treatment for radiation-induced fibrosis, and further studies are urgently needed.

Lipoprotein-associated Phospholipase A2 (Lp-PLA_2_) is a Ca^2+^-independent secreted phospholipase A2 that is primarily synthesized and released by inflammatory cells, including macrophages and mast cells, as well as by endothelial cells [7]. Its enzymatic activity generates pro-inflammatory mediators such as lysophosphatidylcholine (LPC) and oxidized fatty acids, which contribute to the development and progression of atherosclerosis [8]. Elevated Lp-PLA_2_ levels have been detected in the plasma of patients with hypertension and in the pathological tissues of patients with fibrotic diseases [9]. Moreover, radiation exposure increases Lp-PLA_2_ expression, which is associated with radiation-induced inflammation and endothelial dysfunction [10].

Darapladib is a potent oral inhibitor of Lp-PLA_2_ that has been evaluated in phase III clinical trials in patients with atherosclerosis [11] and has recently shown promising therapeutic effects through Lp-PLA_2_ inhibition in diabetic macular edema and Alzheimer’s diseases [12,13]. However, the effect of darapladib on radiation-induced skin injury (RISI) remains unknown. In this study, we investigated the role of darapladib, an Lp-PLA_2_ inhibitor, in radiation-induced endothelial cell damage, inflammation, and fibrosis, and explored the underlying mechanisms and protective effects of darapladib in IRIS.

## 2. Materials and Methods

### 2.1. Animal Experiments

All animal procedures were approved by the Animal Care and Use Committee of the Korea Institute of Radiological and Medical Sciences (KIRAMS). Male SKH1 hairless mice and C57BL/6 mice were purchased from Dooyeol Biotech (Seoul, South Korea) and maintained under specific pathogen-free (SPF) conditions. SKH1 hairless mice were randomly assigned to four groups (*n* = 5 per group): control, irradiation (IR), darapladib (Dara) 20 mg/kg, and darapladib 40 mg/kg. Darapladib (Cayman Chemicals, Ann Arbor, MI, USA) was administered subcutaneously into the dorsal skin of 6-week-old mice 1 day before irradiation with a X-Rad 320 irradiator (Precision X-Ray, Madison, CT, USA). Control mice received the equal volume of DPBS. All SKH1 mice were sacrificed 2 weeks after irradiation. Skin tissues were either fixed in 10% neutral-buffered formalin for histological analysis or processed for RNA and protein extraction. Femurs and tibias from 8-week-old C57BL/6 mice were harvested for the isolation of bone marrow–derived macrophages (BMDMs).

### 2.2. Cell Culture

Human Dermal Microvascular Endothelial Cells (HDMECs) were obtained from PromoCell (Heidelberg, Germany) and cultured in endothelial growth medium MV supplemented with SupplementMix^®^ (#C-22020, PromoCell, Heidelberg, Germany) containing 0.4% (*v*/*v*) ECGS/H, 5.0% (*v*/*v*) FBS, 10 ng/mL EGF, 1 μg/mL hydrocortisone, 100 U/mL penicillin, and 100 μg/mL streptomycin. RAW264.7 murine macrophages were obtained from ATCC (Virginia, MA, USA) and cultured in RPMI-1640 medium supplemented with 10% FBS, 1% penicillin–streptomycin, and 1% Antibiotic-Antimycotic (Gibco, Waltham, MA, USA). Primary human dermal fibroblasts (HDFs) were obtained from ATCC (Virginia, MA, USA) and maintained in DMEM supplemented with 10% FBS, 1% penicillin–streptomycin, and 1% Antibiotic-Antimycotic (Gibco, Waltham, MA, USA). All cells were maintained at 37 °C in a humidified incubator with 5% CO_2_ and used between passages 5 and 10. Cultured cells were exposed to γ-rays from a ^137^Cs source (Biobeam8000, STS Steuerungstechnik & Strahlenschutz GmbH, Braunschweig, Germany) at a dose rate of 2.73 Gy/min. Cells were harvested using trypsin-EDTA and counted with a TC20 automated cell counter (Bio-Rad, Hercules, CA, USA). Harvested cells were washed once with PBS and centrifuged at 500× *g* for 5 min at 4 °C.

### 2.3. Transfection with Small Interfering RNAs (siRNAs)

HDMECs were transfected with either control siRNA or siRNA targeting Lp-PLA_2_ at 60–70% confluency. Transfections were performed in 35-mm dishes using 200 pmol siRNAs per dish, according to the manufacturer’s protocol (Lipofectamine RNAiMAX Transfection Reagent, Invitrogen, Waltham, MA, USA). Forty-eight hours after transfection, cells were lysed in RIPA buffer (50 mM Tris-HCL, pH 7.4, 1% TritonX-100, 150 mM NaCl, 1 mM EDTA, 0.1% SDS) containing 1 mM DTT and 1 mM of Na_3_VO_4_, and proteins of interest were analyzed by Western blotting.

### 2.4. Matrigel In Vitro Endothelial Tube Formation Assay

Endothelial cell tube formation was assessed on Matrigel-coated chamber slides as previously described [14]. Tube networks were photographed using an Eclipse Ts2 microscope (Eclipse Ts2, Nikon Corporation, Minato-ku Tokyo, Japan) at 40× magnification. Tube formation was quantified by counting the number of connected cells in randomly selected fields at 200× magnification with a microscope and dividing this value by the total number of cells in the same field.

### 2.5. Mitochondrial Membrane Potential and Immunofluorescence

Cells were seeded at a density of 5 × 10^3^ cells per well on immunofluorescence microscope slides. After attachment, they were pretreated with either vehicle or darapladib (Cayman Chemicals, Ann Arbor, MI, USA) for 1 h and then exposed to IR. Mitochondrial membrane potential was assessed using the MitoProbe™ JC-1 Assay Kit (M34152, Invitrogen, Carlsbad, CA, USA), according to the manufacturer’s instructions. JC-1-stained cells were examined using a fluorescence microscope (DMi8, Leica Microsystems, Wetzlar, Germany). For immunofluorescence staining, identically treated cells were fixed with 4% paraformaldehyde and permeabilized with 0.5% PBST. Samples were incubated with a primary antibody against phospho-Histone H2AX (γ-H2AX, Ser139; #2577, Cell Signaling Technology, Danvers, MA, USA), followed by fluorescently labeled secondary antibodies. Nuclei were counterstained with DAPI (Invitrogen, Thermo Fisher Scientific, Carlsbad, CA, USA), and images were acquired using a fluorescence microscope.

### 2.6. Western Blotting

For Western blotting, cell lysates were mixed 1:1 (*v*/*v*) with gel-loading buffer (0.125 M Tris-HCl, pH 6.8, 4% SDS, 10% 2-mercaptoethanol, and 0.2% bromophenol blue) and boiled for 5 min. Equal amounts of protein were separated by SDS-PAGE on 10% acrylamide gels and transferred to nitrocellulose membranes at 100 V for 1 h using a wet transfer system. Membranes were blocked in 5% non-fat milk in TBS-T (10 mM Tris, pH 7.5, 100 mM NaCl, 0.1% Tween 20) for 1 h at room temperature and then incubated with primary antibodies at 4 °C overnight. After washing, membranes were incubated for 1 h at 25 °C with horseradish peroxidase (HRP)-conjugated secondary antibodies, including anti-mouse, anti-rabbit (both 1:10,000; Santa Cruz Biotechnology, TX, USA), or anti-goat (1:10,000; Santa Cruz). Antibody binding was visualized using West-zol Plus chemiluminescence and imaged with a FluorChem TMSP system (Alpha Innotech Corporation, San Leandro, CA, USA).

### 2.7. Quantitative Real-Time Polymerase Chain Reaction (Real-Time PCR)

Total RNA was isolated from cells and skin tissues using the MasterPure^TM^ complete RNA Purification Kit (Lucigen) according to the manufacturer’s instructions and reverse transcribed using the ImProm-II reverse transcription system (Promega, Madison, WI, USA). Quantitative RT-PCR was performed on a Chromo 4 Cycler (Bio-Rad, Hercules, CA, USA) using Power SYBR Green PCR Master Mix (Applied Biosystems, Waltham, MA, USA) and gene-specific primers.

### 2.8. Enzyme-Linked Immunosorbent Assay (ELISA)

Oxidized low-density lipoprotein (oxLDL) levels were measured using an Oxidized LDL ELISA kit (Cell Biolabs, Inc., San Diego, CA, USA) according to the manufacturer’s instructions. samples were treated with LDL precipitation solution to remove nonspecific proteins prior to loading. After adding of standards and samples, plates were incubated for 2 h at room temperature, followed by washing and blocking. A biotinylated anti-ApoB-100 antibody was then added to detect captured oxLDL, followed by a streptavidin–HRP conjugate to bind the biotinylated antibody. Total TGF-β levels were quantified using a TGF-β ELISA kit (Abcam, Cambridge, UK), and total LPC levels were using a LPC ELISA kit (#MBS2700657, MyBiosource, Inc., San Diego, CA, USA). A high-binding 96-well plate was coated with capture antibodies and incubated overnight at 4 °C. After two washes with PBST, the plate was blocked for 1 h at room temperature. Standards and acid-activated serum samples were then added. Following incubation with the detection antibody solution for 1 h at 400 rpm and room temperature, plates were treated with enzyme conjugate solution for 30 min at 400 rpm. After thorough washing with PBST, 100 µL of TMB substrate solution was added and incubated for 15–30 min at 37 °C in the dark. The reaction was terminated by adding 50 µL of stop solution, and the absorbance was measured at 450 nm using a microplate reader (Multiskan FC, Thermo Fisher Scientific, Waltham, MA, USA).

### 2.9. Flow Cytometry

Excised skin samples were enzymatically dissociated using a Multi Tissue Dissociation Kit (Multi tissue dissociation kit; Miltenyi Biotec, Bergisch Gladbach, Germany) according to the manufacturer’s instructions. The dissected skin adjacent to the fibrotic region was mechanically dissociated through a 100 µm nylon cell strainer (SPL Life Sciences, Gyeonggi-do, Korea) to obtain single-cell suspensions, which were then washed with running buffer (0.5% bovine serum albumin in PBS). Cells were counted using an automated cell counter. Single cells were stained using Fixable Viability Stain 780 or Fixable Viability Stain 620 (Becton Dickinson, Franklin Lakes, NJ, USA) or zombie NIR Fixable Viability dye (BioLegend, San Diego, CA, USA) for 15 min at room temperature, followed by incubation with anti-CD16/32 antibodies (101301, Thermo Fischer Scientific, MA, USA) for 15 min at room temperature. Cells were then placed on ice and surface-labeled with monoclonal antibodies (Table 1) for 20 min. Flow cytometric data were acquired using a CytoFLEX flow cytometer (Beckman Coulter, Brea, CA, USA) and FACSCanto^TM^ (BD Biosciences, NJ, USA). All flow cytometric data were analyzed using Kaluza Analysis 2.0 software (Beckman Coulter, Brea, CA, USA) and FlowJo 10.9. software (Becton Dickinson, Franklin Lakes, NJ, USA).

### 2.10. BMDMs Culture and Reagents

Murine BMDMs were generated from 6–9-week-old female C57BL/6 mice as previously described. Isolated bone marrow cells were cultured for 7 days at a density of ×10^6^ cells/mL in RPMI medium (Thermo Fisher Scientific, Waltham, MA, USA) supplemented with 10% FBS (Gibco, Carlsbad, CA, USA), 10 U/mL penicillin, 10 μg/mL streptomycin and 2 mM L-glutamine (Gibco, Carlsbad, CA, USA) at 37 °C in a humidified atmosphere with 5% CO_2_. To enrich the macrophage population, cells were differentiated with 20 ng/mL GM-CSF for 7 days [15]. For polarization, 20 ng/mL of IFN-γ or TGF-β was added for an additional 2 days [16]. Darapladib was administered throughout the differentiation period. For cytokine response assays, macrophages were stimulated with 20 μg/mL LPC for 24 h.

### 2.11. Measurement of Skin Barrier Function

Skin hydration was evaluated using a Corneometer CM825 (Courage + Khazaka electronic GmbH, Köln, Germany), which measures skin surface moisture based on capacitance and detects water content within a constant depth (10–20 μm) below the stratum corneum. The transepidermal water loss (TEWL) was assessed using a Tewameter TM300 (Courage + Khazaka electronic GmbH, Köln, Germany) to evaluate changes in skin barrier function. The probe was placed perpendicularly on the test site, and TEWL was recorded once the skin had stabilized; higher values indicate greater water loss. All measurements were performed after 30 min of acclimatization under controlled temperature and humidity. Skin surface pH, an indicator of hydrolipid film quality and a regulator of the antimicrobial barrier, permeability barrier homeostasis, and barrier integrity/cohesion, was measured using a Skin pH Meter^®^ PH 905 (Courage + Khazaka electronic GmbH, Köln, Germany). This device uses a flat-topped glass electrode connected to a voltmeter, specifically designed for determining the pH of the stratum corneum.

### 2.12. Hematoxylin and Eosin (H&E) and Masson’s Trichrome (MT) Staining

H&E staining was performed to evaluate the skin architecture and dermal thickness. After deparaffinization and rehydration, slides were rinsed with tap water and stained with Mayer’s hematoxylin solution (S3309, Dako, Glostrup, Denmark) for 1 min, followed by rinsing in tap water. Slides were then treated with 0.1% ammonia solution, counterstained with eosin (#HT110132, Sigma-Aldrich, St. Louis, MO, USA) for 5 min, dehydrated, and coverslipped with neutral gum. Stained sections were examined by light microscope. MT staining was performed using a commercial kit (#25088-1, Polysciences, Warrington, PA, USA) according to the manufacturer’s protocol. Briefly, Paraffin sections were deparaffinized, rehydrated through graded alcohols, and mordanted in pre-warmed Bouin’s solution at 60 °C for 1 h. After rinsing in running water, nuclei were stained with Weigert’s iron hematoxylin (5 min), followed by Biebrich Scarlet-Acid Fuchsin staining for 5 min and differentiation in phosphomolybdic-phosphotungstic acid for 20 min. Collagen fibers were stained with aniline blue for 20 min, after which sections were rinsed in 1% acetic acid for 1 min, dehydrated through graded ethanol, cleared in xylene, and mounted.

### 2.13. Immunohistochemistry (IHC)

IHC was performed using a general IHC detection kit (IHC-761, General Bioscience, Brisbane, CA, USA) according to the manufacturer’s instructions. Primary antibodies included Lp-PLA_2_ (LS-C163765, LS Bio, Newark, CA, USA), inducible nitric oxide synthase (iNOS; sc-7271, Santa Cruz, Dallas, TX, USA), α-SMA (#19245, Cell Signaling, Danvers, MA, USA), and vimentin (NBP1-31327, Novus Biologicals, Centennial, CO, USA). After incubation with the primary and secondary antibodies, slides were developed with a mixed DAB A/B solution for 5 min and rinsed with tap water. Sections were counterstained with hematoxylin (S3309, Dako, Glostrup, Denmark), washed with distilled water, and examined under a light microscope (DMi8, Leica Microsystems, Wetzlar, Germany).

### 2.14. Statistical Analysis

Statistical analyses were performed using GraphPad Prism 10 (GraphPad Software, San Diego, CA, USA). A two-tailed Student’s *t*-test was used to assess differences between two groups, and one-way ANOVA followed by Tukey’s post hoc test was applied for comparisons among multiple groups. Differences were considered statistically significant at *p* < 0.05. Data are presented as mean ± SEM.

## 3. Results

### 3.1. Lp-PLA_2_ Inhibition by Darapladib Ameliorates Radiation-Induced Endothelial Cell Damage

**Figure 1:**
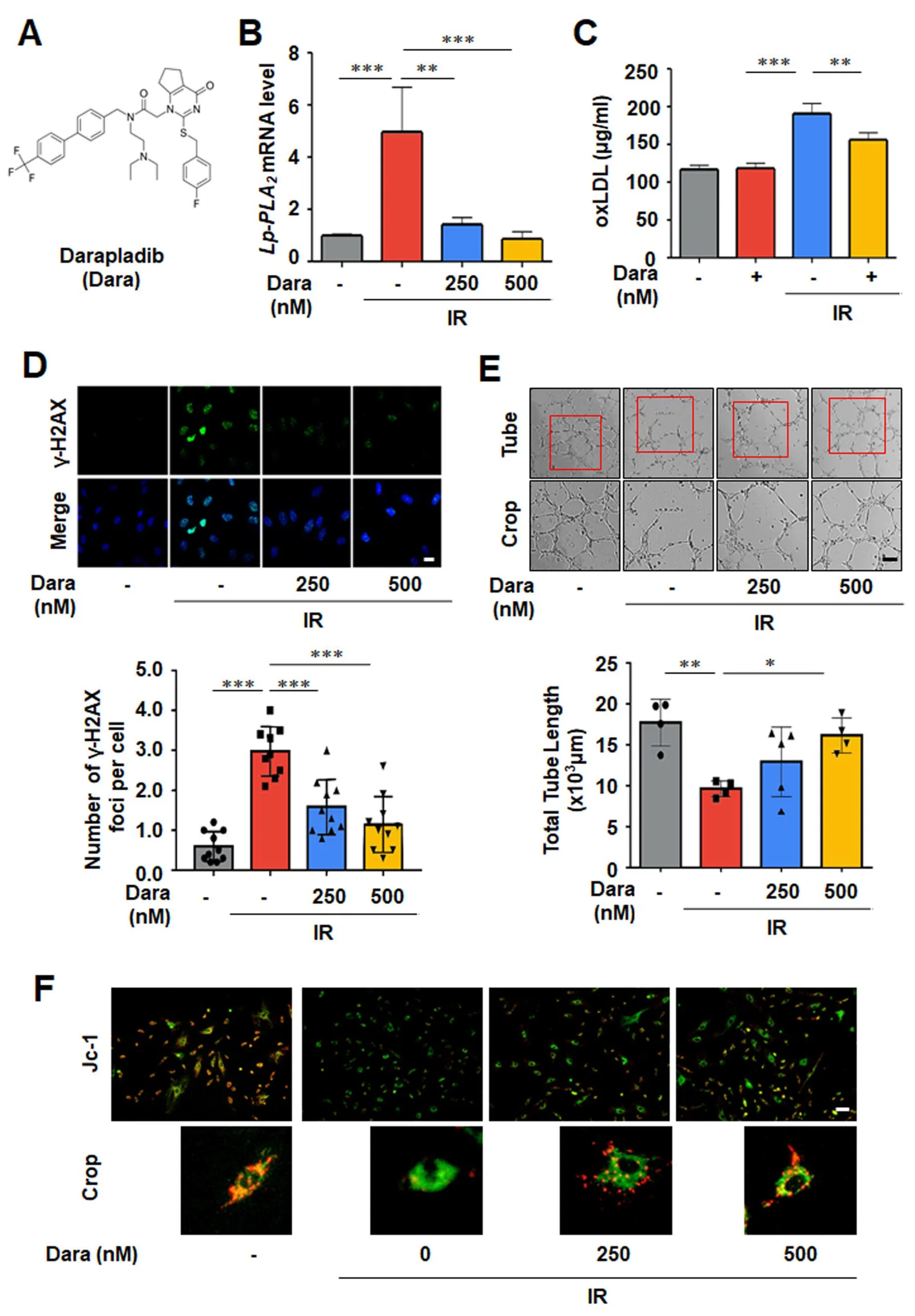
Lp-PLA_2_ inhibition by darapladib ameliorates radiation-induced endothelial cell damage. For all experiments, HDMVECs were pretreated with darapladib before irradiation. (**A**) Chemical structure of darapladib, an Lp-PLA_2_ inhibitor. (**B**) HDMVECs were treated with 0, 250, or 500 nM darapladib 1 h before 5 Gy irradiation. After 24 h, Lp-PLA2 mRNA levels were measured by real-time PCR. (**C**) Cells were treated with 500 nM darapladib 1 h before 5 Gy irradiation and cultured for 2 days. Ox LDL levels were quantified by ELISA. (**D**) Cells were treated with 0, 250, or 500 nM darapladib 1 h before 5 Gy irradiation. After 1 h, cells were immunostained with an anti-γ-H2AX antibody (green) and DAPI (blue). γ-H2AX foci were quantified using a confocal fluorescence microscope (40×). Scale bar = 25 μm. (**E**) Tube formation assays were performed by treating HDMVECs with darapladib 1 h before 5 Gy irradiation. Tube morphology was evaluated 24 h after IR using an In Cell analyzer. Red boxes indicate the enlarged areas. Scale bar = 200 μm. (**F**) HDMVECs were stained with JC-1 dye for 30 min and examined using a confocal fluorescence microscope (10×). Green and red fluorescence indicate low and high mitochondrial membrane potential, respectively. Scale bar = 100 μm. Data are presented as mean ± SEM (*n* = 3–4 independent experiments). Statistical significance was assessed by one-way ANOVA with Tukey’s post hoc test. * *p* < 0.05, ** *p* < 0.01, *** *p* < 0.001 versus the irradiated group.

Darapladib is a structurally defined small molecule composed of a cyclopenta-pyrimidine ring, a fluorobenzyl sulfane moiety, a diethylpropan-amine moiety, and a trifluoromethyl-biphenyl moiety, which enables direct docking to Lp-PLA_2_ (Figure 1A) [17]. Darapladib significantly suppressed IR-induced Lp-PLA_2_ mRNA expression. (Figure 1B). In our previous study, we demonstrated that radiation exposure induces the production of oxLDL and Reactive oxygen species (ROS), which in turn activate Lp-PLA2 and trigger a cascade of endothelial cell-damaging events [10]. Consistent with this, Darapladib treatment reduced oxLDL levels and intracellular ROS following irradiation (Figure 1C). To evaluate the protective effects of darapladib on endothelial cells after radiation exposure, we quantified γ-H2AX foci as a marker of DNA double-strand breaks and performed tube formation assay to assess endothelial function. IR-induced γ-H2AX phosphorylation was reduced by darapladib treatment. In the IR group, the tube-forming capacity of endothelial cells on Matrigel was significantly impaired, but darapladib restored tube formation to levels comparable to the control group (Figure 1D,E). Because mitochondrial dysfunction is a key feature of radiation-induced oxidative injury in endothelial cells, we assessed mitochondrial membrane potential using the JC-1 probe. Darapladib-treated cells exhibited increased red fluorescence and decreased green fluorescence, indicating recovery of mitochondrial function following irradiation (Figure 1F). Together, these results demonstrate that darapladib alleviates radiation-induced cellular damage and endothelial dysfunction via Lp-PLA_2_ inhibition.

### 3.2. Lp-PLA_2_ Regulates Radiation-Induced Transdifferentiation

**Figure 2:**
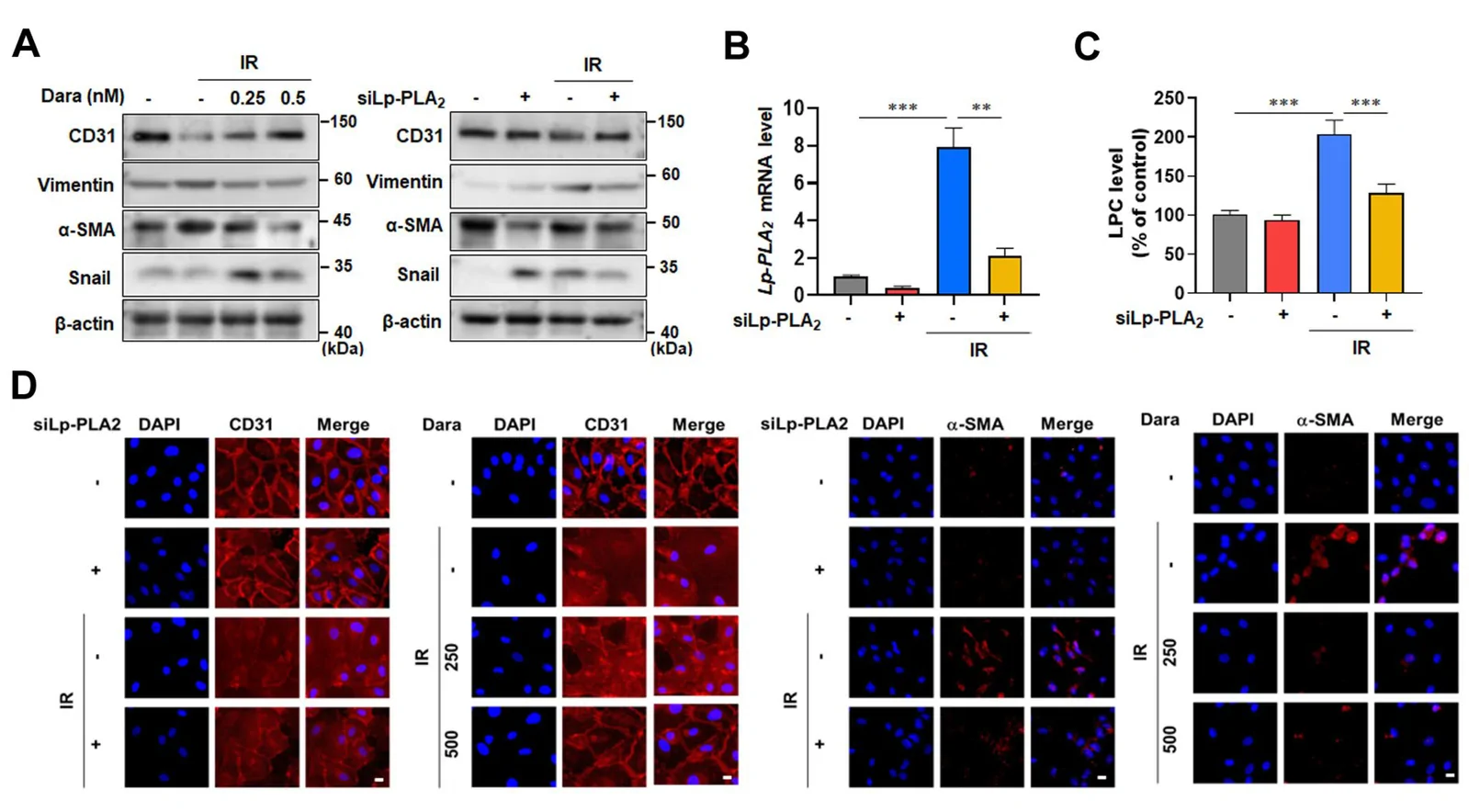
Lp-PLA_2_ regulates radiation-induced EndoMT in HDMVECs. (**A**) HDMVECs were treated with 0, 250, or 500 nM darapladib 1 h before 5 Gy irradiation. After 24 h, Vimentin, Snail and α-SMA levels were analyzed by Western blotting. In parallel, cells were transfected with 200 pmole of Lp-PLA_2_ siRNA for 2 days and then exposed to 5 Gy IR for 24 h. Proteins were extracted and immunoblotted with the same antibodies. β-actin served as a loading control. (**B**) Cells were transfected with 200 pmole of Lp-PLA_2_ siRNA for 2 days, and Lp-PLA_2_ mRNA level was quantified by real-time PCR. (**C**) Lp-PLA2 siRNA-transfected cells were treated with IR for 48 h, and LPC generation was analyzed using ELISA. ** *p* < 0.01, *** *p* < 0.001 versus the IR group. (**D**) Cells were stained with anti-CD31 or anti-a-SMA antibodies and examined by confocal microscope. Blue represents DAPI staining, and red represents CD31 or α-SMA staining. Scale bar = 25 μm.

**Figure 3:**
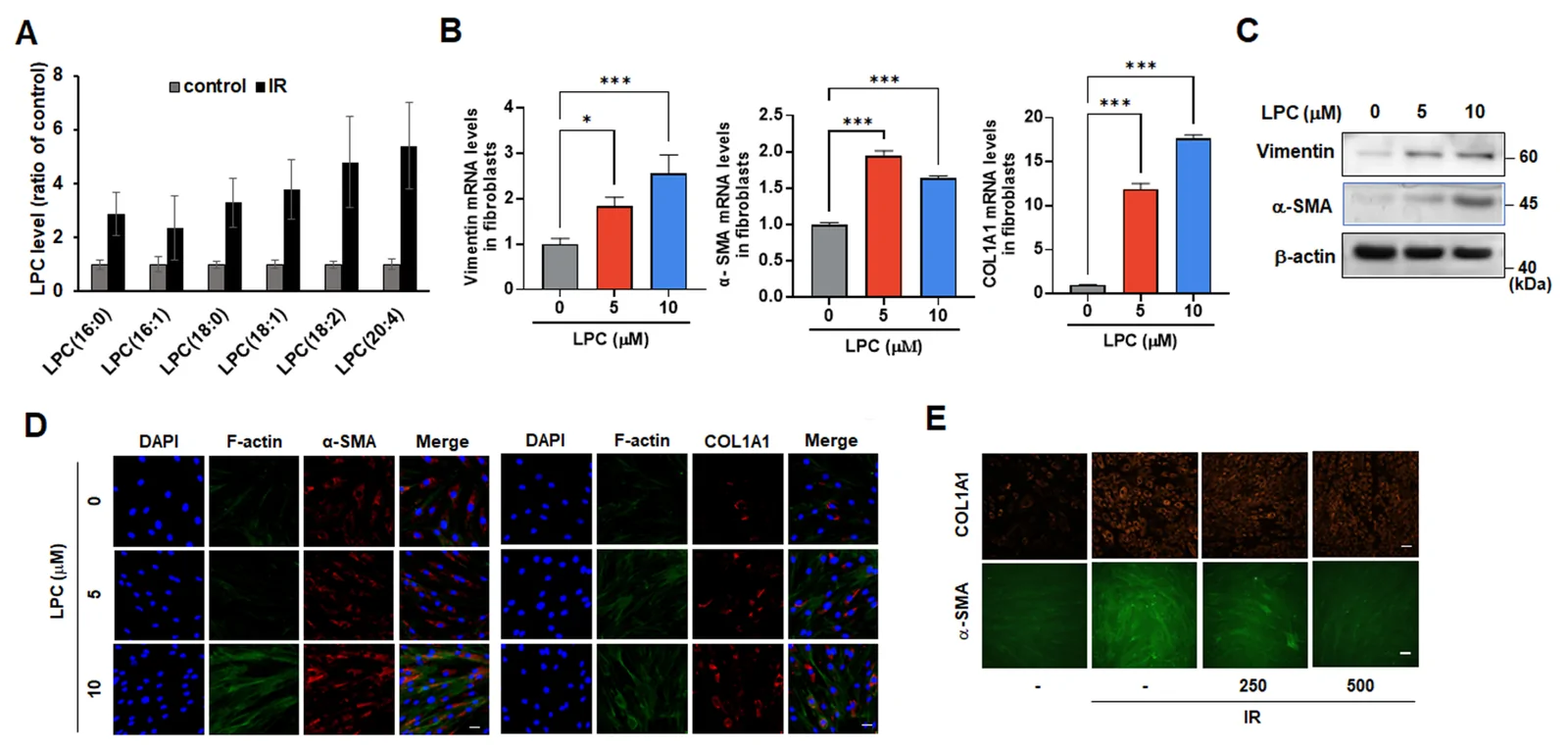
Radiation-induced LPC promotes fibroblast-to-myofibroblast transition. (**A**) HDFs were irradiated with 5 Gy and harvested at 48 h after irradiation. Cell lysates were analyzed for LPC species by UPLC-ESI-QTOF MS. (**B**) HDFs treated with 0, 5, or 10 μM LPC (18:0) for 48 h. Vimentin, α-SMA and β-actin levels were assessed by Western blotting. (**C**) mRNA was extracted from HDFs treated as in (**B**) and relative mRNA expressions of Vimentin, α-SMA and Col1A1 were analyzed using real-time PCR. Data represent target gene expression normalized to a GAPDH gene. (**D**) HDFs were treated with 0, 5, or 10 μM LPC (18:0) for 24 h and then stained for F-actin (green) and α-SMA or Col1A1 (red). Nuclei were counterstained with DAPI (blue). (**E**) HDFs were treated with 0, 250, or 500 nM darapladib 1 h before 5 Gy irradiation. After 24 h, the cells were stained for α-SMA (green) and Col1A1 (red). Scale bar = 25 μm. * *p* < 0.01, *** *p* < 0.001 versus control.

Radiation exposure induces EndoMT and vascular injury, leading to tissue damage [18]. To investigate the involvement of Lp-PLA_2_ in radiation-induced EndoMT, HDMVECs were treated with darapladib or transfected with Lp-PLA_2_ siRNA (Figure 2A,B). Lp-PLA_2_ depletion inhibited radiation-induced LPC production (Figure 2C). In irradiated HDMVECs, expression of CD31, an endothelial marker, was reduced, whereas the expression of fibroblast markers, including α-SMA, Snail, and Vimentin, was increased (Figure 2A,D). Inhibition of Lp-PLA_2_ by either siRNA or darapladib suppressed these radiation-induced phenotypic changes, restoring CD31 expression and attenuating the induction of α-SMA, Snail and Vimentin (Figure 2A,D). LPC analysis of irradiated human fibroblasts using UPLC-ESI-QTOF mass spectrometry was performed, and radiation promoted production of multiple LPC species in fibroblasts (Figure 3A). To assess whether LPC contributes to fibroblast-to-myofibroblast transition (FMT), we treated fibroblasts with LPC (18:0), a species implicated in endothelial damage [10]. LPC-induced expression of FMT markers α-SMA and vimentin (Figure 3B,C) generated stress fibers and increased collagen expression and secretion (Figure 3C,D). Lp-PLA_2_ inhibition by darapladib treatment decreased expression of FMT markers α-SMA and vimentin in fibroblasts (Figure 3E). Together, these data indicate that LPC promotes transdifferentiation across multiple stromal cell types, and that inhibition of Lp-PLA_2_ attenuates radiation-induced FMT as well as EndoMT, limiting the acquisition of pro-fibrotic phenotypes.

### 3.3. Darapladib Inhibits Radiation-Induced Skin Injury and Restores Barrier Integrity via Lp-PLA_2_ Inhibition

**Figure 4:**
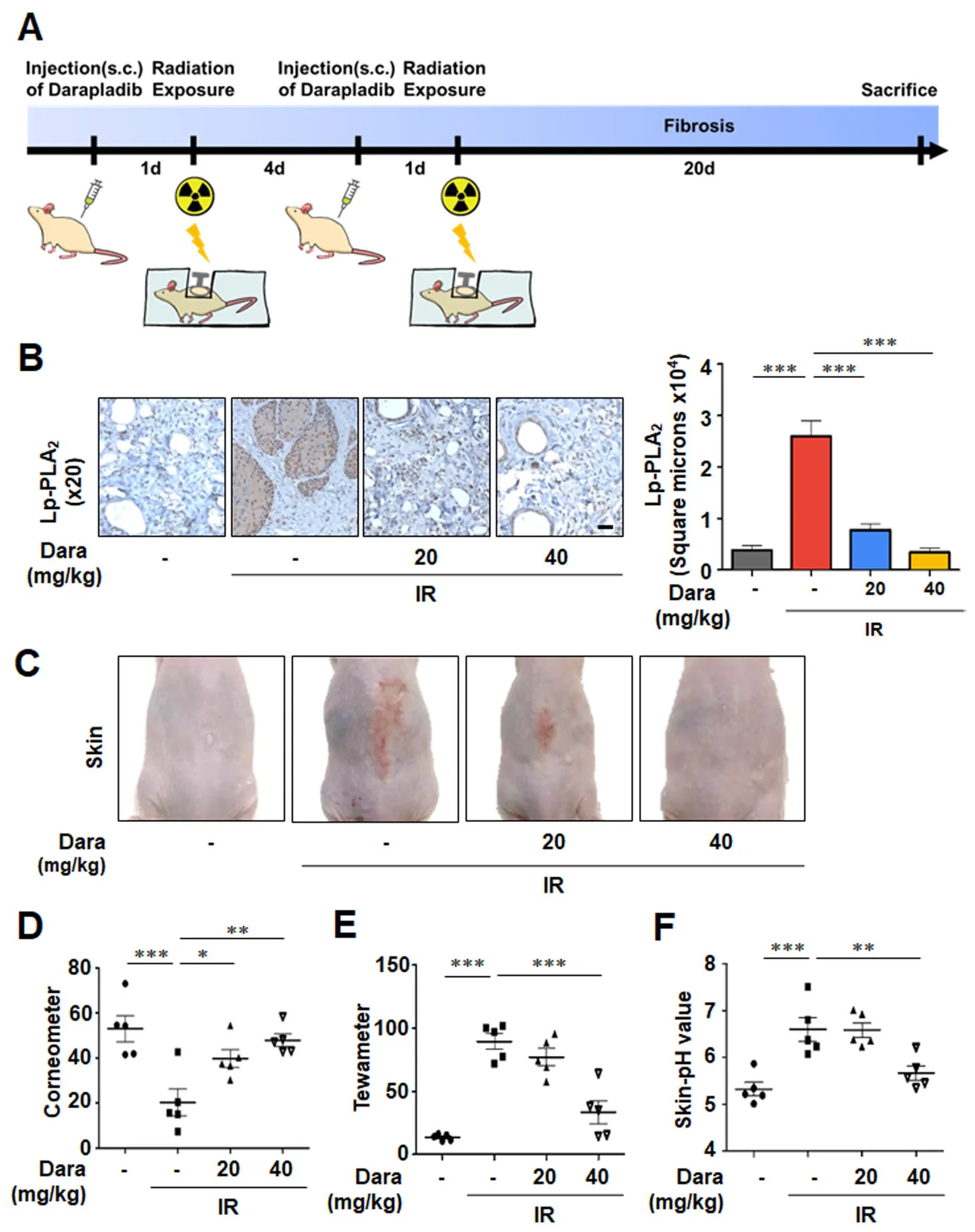
Darapladib inhibits radiation-induced skin injury and promotes restoration of barrier integrity. (**A**) Schematic representation of the mouse model of RISI and fibrosis. Mice received subcutaneous injections of 20 or 40 mg/kg darapladib 1 day before IR. A total of two injections were administered within 1 week. (**B**) IHC staining of Lp-PLA_2_ in radiation-induced scarred skin was performed 3 weeks after irradiation. Lp-PLA_2_-positive areas were quantified as the mean value from five fields per section. Scale bar = 50 μm. (**C**) Representative images of mouse skin showing radiation-induced scar lesions and their attenuation by darapladib treatment (*n* = 5). Skin hydration (**D**), TEWL (**E**), and surface pH (**F**) were measured in scar lesional and adjacent normal skin. Measurements for each animal were repeated until stable and consistent values were obtained (*n* = 5). Data are presented as mean ± SEM. Statistical significance was determined by one-way ANOVA with Tukey’s post hoc test. *n* = 3–4 independent experiments. * *p* < 0.05, ** *p* < 0.01, *** *p* < 0.001 versus the IR group.

To assess the effect of darapladib on RISI, SKH1 mice received two single doses of 20 Gy local IR over 20 days and were treated with two subcutaneous injections of darapladib (20 or 40 mg/kg) into the dorsal skin (Figure 4A). In irradiated mice, Lp-PLA_2_ expression was increased in skin sections, whereas darapladib significantly reduced Lp-PLA_2_ levels (Figure 4B). Radiation-induced skin scarring, a common adverse effect of radiation therapy, was clearly evident in the IR group but was markedly attenuated in darapladib-treated mice. Notably, the 40 mg/kg dose almost nearly abolished visible scarring (Figure 4C). Because RISI disrupts moisture and pH balance, leading to excessive moisture loss, we next evaluated skin barrier function. In irradiated mice, skin hydration measured by corneometer was decreased, whereas darapladib treatment dose-dependently restored moisture content to near control levels (Figure 4D). TEWL was elevated in the IR group but was significantly reduced by 40 mg/kg darapladib (Figure 4E). In control mice, skin pH was maintained at approximately 5.5. Following radiation exposure, skin pH increased to 7–8, consistent with dermatitis-like barrier disruption. Notably, darapladib treatment restored skin pH to near-control levels (pH 5–6) (Figure 4F). Taken together, these findings indicate that darapladib effectively attenuates radiation-induced skin injury and restores skin barrier function by normalizing both moisture content and pH levels.

### 3.4. Darapladib Attenuates Radiation-Induced Skin Fibrosis

**Figure 5:**
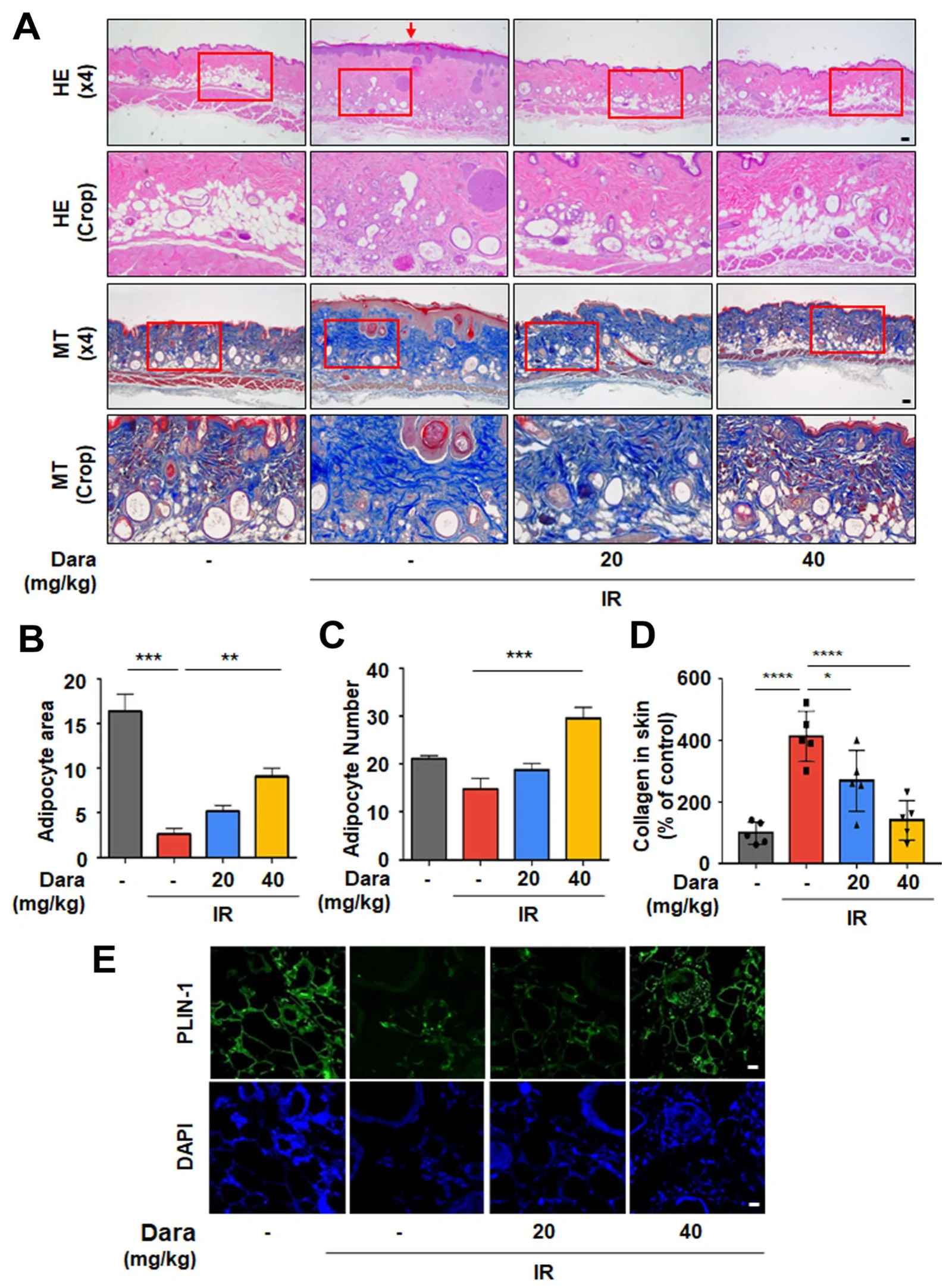
Darapladib attenuates radiation-induced skin fibrosis. (**A**) H&E and MT-stained images in skin tissues were generated 3 weeks after irradiation, with or without darapladib treatment. Red boxes indicate the enlarged areas. Scale bar = 100 µm. (**B**,**C**) The quantification of adipocyte number and area was measured per field, averaged over five fields in H&E images. (**D**) The quantification of collagen deposition area per field was averaged from five fields in MT images. (**E**) Immunofluorescence staining of PLIN-1 (green) was analyzed in skin tissues from non-irradiated and irradiated mice, with or without darapladib treatment. Scale bar = 25 µm. Data are presented as mean ± SEM (*n* = 3–4 independent experiments). Statistical significance was determined by one-way ANOVA with Tukey’s post hoc test. * *p* < 0.05, ** *p* < 0.01, *** *p* < 0.001, **** *p* < 0.0001 versus the IR group.

To determine whether improvements in fibrosis and barrier function were reflected in deeper tissue architecture, histological analyses of the stratum corneum, epidermis, dermis, and subcutaneous layer were performed (Figure 5A). Radiation induced detachment of the stratum corneum (arrow), compromising skin hydration, whereas darapladib preserved stratum corneum integrity. Compared with controls, irradiated skin showed increased epidermal and dermal thickness, which was reduced by darapladib treatment. To assess radiation-induced lipid loss, changes in dermal white adipose tissue (DWAT) were quantitatively evaluated by H&E staining of skin cross-sections. Adipocyte content was markedly decreased in irradiated skin but was restored by darapladib (Figure 5B,C). MT staining demonstrated that radiation-induced collagen deposition was reduced by darapladib in a dose-dependent manner, (Figure 5D). Consistent with these observations, immunofluoresnce analysis of PLIN-1, a lipid droplet-associated protein, showed that the lipid composition in irradiated skin was restored following darapladib treatment (Figure 5E).

### 3.5. Darapladib Suppresses TGF-β Signaling in Radiation-Induced Skin Fibrosis In Vivo

**Figure 6:**
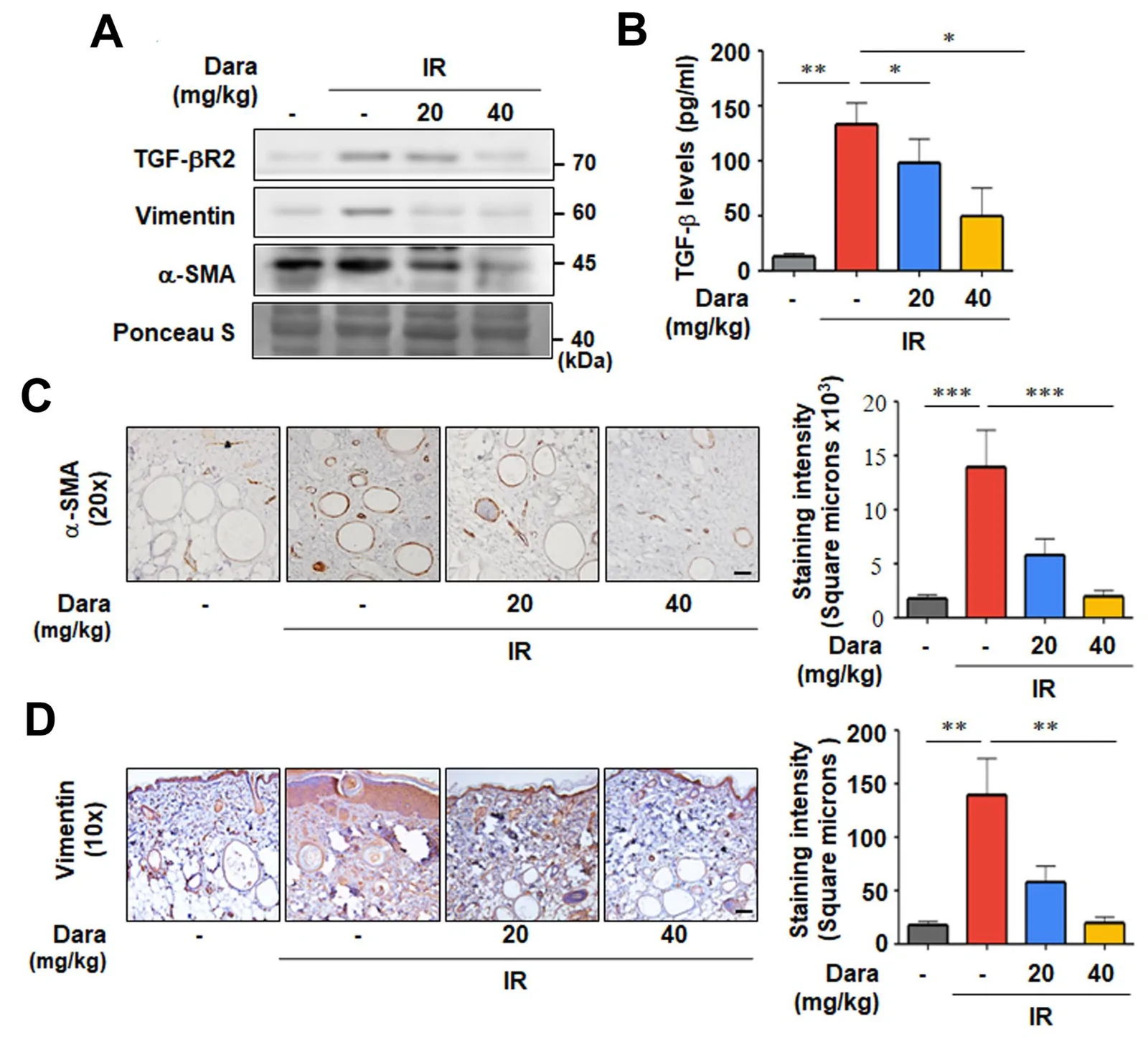
Darapladib suppresses TGF-β signaling in radiation-induced skin fibrosis in vivo. (**A**) Proteins were extracted from mouse skin 4 weeks after irradiation. TGF-βR2, Vimentin and α-SMA levels were analyzed by Western blotting, using ponceau S staining as a control. (**B**) TGF-β levels were measured using ELISA. Immunohistochemical staining of α-SMA (**C**) and vimentin (**D**) in skin tissues was analyzed 4 weeks after irradiation. Scale bars = 50 µm (**C**) and 100 µm (**D**). Data are presented as mean ± SEM (*n* = 3–4 independent experiments). Statistical significance was determined by one-way ANOVA with Tukey’s post hoc test. * *p* < 0.05, ** *p* < 0.01, *** *p* < 0.001 versus the IR group.

As shown at the cellular level in Figure 2, we next examined whether darapladib could inhibit fibrotic processes such as EndoMT in irradiated mouse skin by assessing the expression of EndoMT-related proteins. As expected, darapladib treatment reduced TGF-β receptor protein levels and suppressed radiation-induced increases in α-SMA and Vimentin (Figure 6A). Because TGF-β is a key signaling mediator in radiation-induced fibrosis [19], we investigated whether darapladib modulates TGF-β expression in irradiated skin. TGF-β levels were elevated in the IR group, whereas darapladib attenuated this radiation-induced upregulation, suggesting an inhibitory effect on fibrosis and EndoMT (Figure 6A,B). IHC analysis further revealed prominent α-SMA expression around DWAT in irradiated skin, which was markedly reduced by darapladib (Figure 6C) In addition, radiation-induced Vimentin accumulation in the epidermis was effectively inhibited by darapladib treatment (Figure 6D). Together, these findings suggest that darapladib attenuates fibrosis by suppressing fibrotic processes, including EndoMT and TGF-β signaling.

### 3.6. Darapladib Reduces Inflammatory Cell Infiltration and Regulates Macrophage Polarization

**Figure 7:**
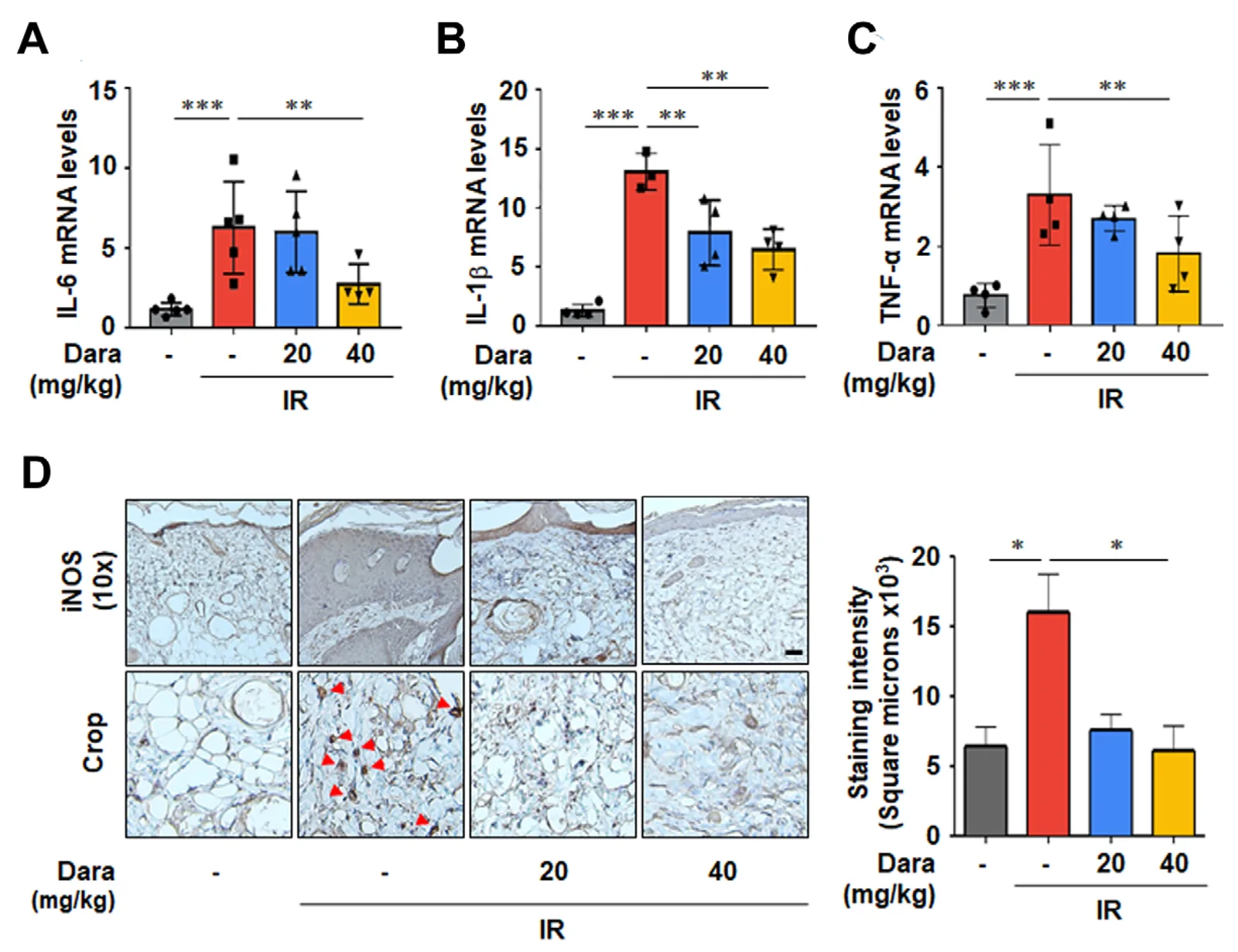
Darapladib reduces radiation-induced late-stage inflammatory cytokine expression in mouse skin. mRNA was extracted from irradiated mouse skin 3 weeks after IR. Relative mRNA expressions of IL-6 (**A**), IL-1β (**B**) and TNF-α (**C**) were analyzed using real-time PCR. Data represent target gene expression normalized to a GAPDH gene. (**D**) IHC staining of iNOS expression in skin tissues was examined 3 weeks after irradiation. Scale bar = 100 μm. Data are presented as mean ± SEM (*n* = 3–4 independent experiments). Statistical significance was determined by one-way ANOVA with Tukey’s post hoc test. * *p* < 0.05, ** *p* < 0.01, *** *p* < 0.001 versus the IR group.

**Figure 8:**
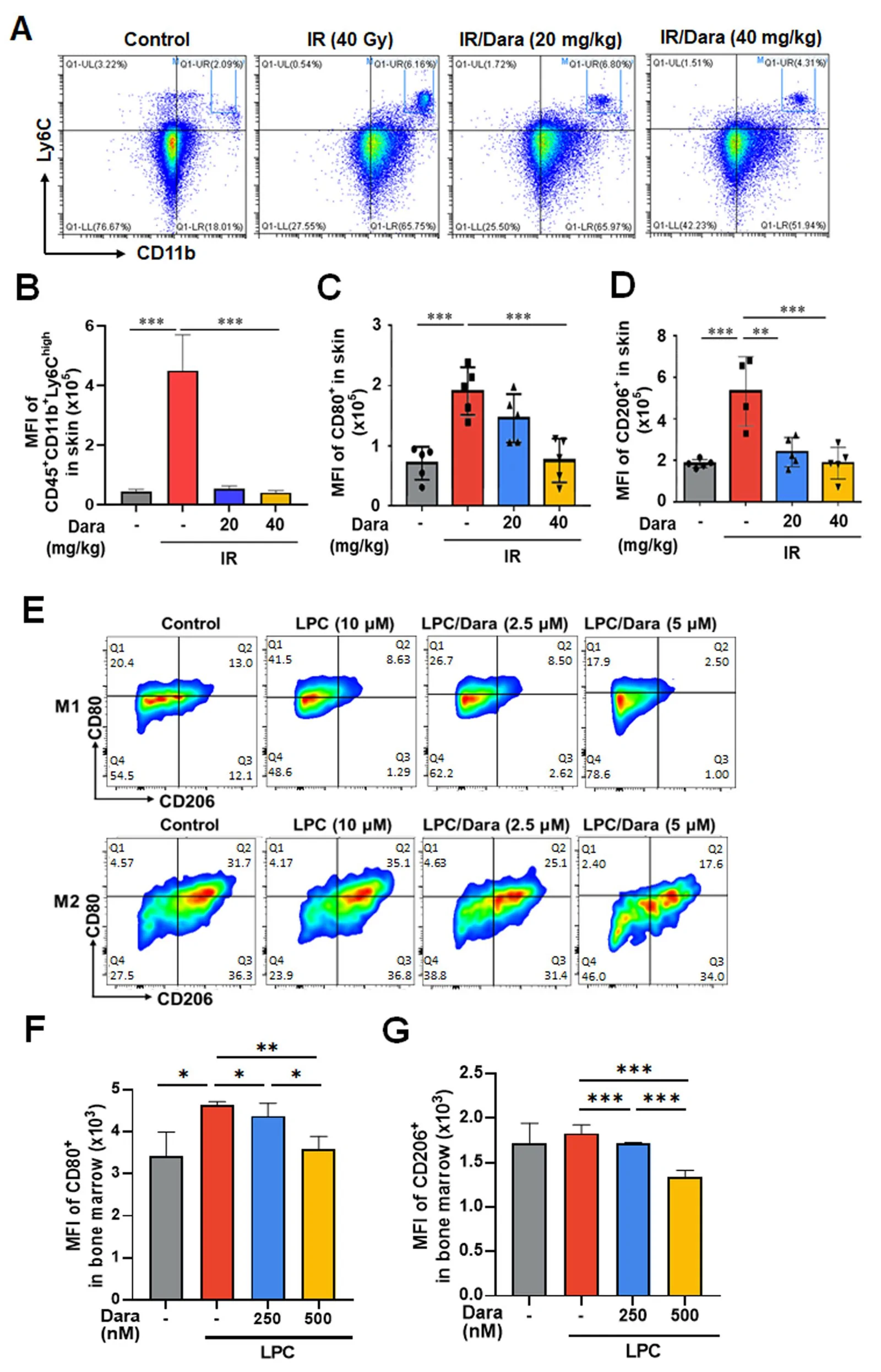
Darapladib reduces radiation-induced macrophage infiltration and inflammatory polarization in mouse skin. (**A**,**B**) Mice were locally irradiated (40 Gy) to the skin and treated with darapladib (20 or 40 mg/kg). Skin tissues were collected and analyzed for Ly6C^high^ inflammatory macrophages by flow cytometry. In the mouse skin tissues, the number of CD80^+^ M1 macrophages (**C**) and CD206^+^ M2 macrophages (**D**) were analyzed by flow cytometry. (**E**) Bone marrow-derived macrophages (BMDMs) were polarized with 20ng/mL IFN-γ (**F**) or 20 ng/mL TGF-β treatment for 2 days (**G**). Differentiated BMDMs were then stimulated with 20 μg/mL lysophosphatidylcholine (LPC) and treated with darapladib for 24 h. The expression of CD80 and CD206 was analyzed by flow cytometry. Data are presented as mean ± SEM. Statistical significance was determined by one-way ANOVA with Tukey’s post hoc test. * *p* < 0.05, ** *p* < 0.01, *** *p* < 0.001. UMI; Unique molecular identifier.

Pro-inflammatory cytokines, including IL-6, IL-1β, and TNF-α, were reduced decreased in the skin of darapladib-treated groups compared to the irradiated group (Figure 7A–C). The iNOS pathway is activated by pro-inflammatory stimuli such as cytokines and promotes inflammation by generating citrulline and nitic oxide from arginine [20]. IHC revealed dense infiltration of iNOS-positive cells in irradiated skin (red triangle), which was markedly decreased by darapladib treatment (Figure 7D). Taken together, these data suggest that darapladib effectively suppresses radiation-induced inflammation. To further investigate the effects of darapladib on macrophage subsets, cells were isolated from lesional mouse skin 14 days after irradiation and analyzed by flow cytometry. Infiltration of CD11b^+^Ly6C^high^ inflammatory macrophages was increased in irradiated skin, while darapladib treatment reduced this population (Figure 8A,B). We next examined macrophage polarization by analyzing M1- and M2-like subsets using CD80 and CD206 as surface markers, respectively. The proportions of both M1- and M2-like macrophages were elevated in irradiated skin compared with controls, and darapladib significantly decreased both populations (Figure 8C,D). To determine whether darapladib directly affects M1/M2 macrophage differentiation, BMDMs were isolated from mice and cultured in vitro. For M1 polarization, bone marrow cells were treated with 20 ng/mL IFN-γ for 2 days. As shown in Figure 8E,F, LPC stimulation increased CD80 expression in M1-like macrophages, whereas darapladib treatment significantly reduced CD80 expression. Similarly, for M2 polarization, bone marrow cells were treated with 20 ng/mL TGF-β for 2 days. LPC slightly increased CD206 expression in M2-like macrophages, but darapladib significantly decreased CD206 levels (Figure 8E,G). These findings indicate that darapladib attenuates M1 and M2 macrophage infiltration during radiation-induced skin injury, potentially through the direct regulation of macrophage differentiation. Together, these results support the translational potential of Lp-PLA_2_ inhibition as a therapeutic strategy for chronic fibrotic diseases by targeting macrophage-mediated inflammation.

## 4. Discussion

RT is an indispensable component of treatment for many malignancies. However, a major adverse effect of RT is radiation-induced-dermatitis and fibrosis, with moderate to severe skin reactions occurring in up to 95% of patients [21]. Skin fibrosis can delay oncologic treatment schedules and markedly impair quality of life, yet effective therapeutic and preventive strategies for radio-dermatitis and fibrosis remain limited. For the development of fibrosis-targeted therapies, it is crucial to concurrently suppress inflammation and provide early vascular protection. The nature of the inflammatory response strongly influences resident tissue cells and infiltrating immune cells, which in turn amplify inflammation through the secretion of chemokines, cytokines, and growth factors. Numerous cytokines participate in wound healing and fibrogenesis, with distinct gene expression programs activated under different conditions. Patients with fibrotic disease frequently exhibit pro-inflammatory cytokine profiles characterized by elevated IL-1α, IL-1β, TNFα, TGFβ, and platelet-derived growth factors (PDGF) [22]. Each of these cytokines can exhibit significant pro-fibrotic activity, acting through the recruitment, activation, and proliferation of fibroblasts, macrophages, and myofibroblasts [23].

In this study, we show that darapladib, an Lp-PLA2 inhibitor, attenuates radiation-induced stromal cell alterations and skin injury. Vascular injury and endothelial dysfunction are early triggers of fibrosis and may be initiated by autoantibodies, chemical insults or oxidative products [24]. Because Lp-PLA_2_ hydrolyses oxidized phospholipids to generate several LPCs and oxidized free fatty acids, radiation-induced upregulation of Lp-PLA2 increased LPC levels (Figure 2). LPC, a major phospholipid of oxidized LDL, has been implicated in radiation-mediated endothelial injury [10] and in functional alterations of smooth muscle cells, fibroblast-like cells and macrophages [25,26,27]. Consistent with this, darapladib reduced radiation-induced DNA damage in endothelial cells (Figure 1) and inhibited transdifferentiation of endothelial cells, fibroblasts and macrophages, implicating LPC in EndoMT and FMT (Figure 2 and Figure 3). Given recent evidence that metabolic reprogramming underlies radiation-induced fibrosis through myofibroblast activation and excessive extracellular matrix deposition [28], LPC may promote fibrosis via metabolic reprogramming and mitochondria-dependent collagen production [29,30]. Darapladib therefore represents a potential therapeutic strategy to interrupt these LPC-mediated pro-fibrotic mechanisms.

Myofibroblasts are recognized as key effector cells in fibrogenesis [31]. They are characterized by contractile activity and distortion of tissue architecture due to their expression of α-SMA [32]. Their origin has been linked to the activation of local stromal cells, such as fibroblasts and pericytes, in the presence of pro-fibrotic factors [33], as well as to transdiffentiation, whereby cells exposed to TGF-β acquire mesenchymal properties [34,35]. In radiation-injured skin, darapladib treatment also reduced the expression of key radiation-induced cytokines, including IL-1β, IL-6, TNF-α, and TGF-β (Figure 7). In vivo, darapladib reversed EndoMT and FMT by reducing TGF-β expression, thereby decreasing radiation-induced collagen deposition and restoring radiation-depleted PLIN-1 (Figure 5). These findings suggest that darapladib exerts anti-fibrotic effects by modulating TGF-β-induced differentiation of fibroblasts into myofibroblasts.

Lp-PLA_2_ inhibition markedly suppresses the recruitment and differentiation of fibrogenic macrophages during radiation-induced dermatitis and fibrosis (Figure 8). The extent and severity of tissue damage and fibrosis correlate closely with the degree of macrophage infiltration [36]. Classically activated M1 macrophages release pro-inflammatory cytokines that exacerbate tissue injury, amplify the inflammatory response, and promote myofibroblast formation and fibrocyte accumulation. A growing body of evidence highlights the importance of cellular metabolism in regulation of macrophage polarization. Arginine is particularly critical, as two opposing pathways—the iNOS pathway and the arginase pathway—are associated with M1 and M2 polarization, respectively. Activation of the iNOS pathway generates citrulline and nitric oxide (NO) from arginine, promoting M1 macrophage differentiation, whereas activation of arginase generates ornithine and urea from arginine, promoting M2 macrophage differentiation [37]. M1 macrophages produce cytokines that activate myofibroblasts by inducing pro-inflammatory mediators such as TNF-α, IL-1β, and various chemokines. Therefore, inhibition of M1 macrophage polarization can attenuate tissue remodeling and ameliorate fibrosis [38]. When the acute inflammatory phase subsides, Th2 cytokines are produced and promote the polarization toward the M2 macrophage phenotype [36]. M2 macrophages play a key pro-fibrotic role and secrete large amounts of pro-fibrotic mediators such as TGF-β [39]. They contribute to the resolution and remodeling phase of wound healing and tissue repair by releasing IL-4, arginase, and TGF-β. In addition, M2 macrophages can activate resident fibroblasts through the production of TGF-β, FGF, VEGF, and galactin-3 [40]. Collectively, this evidence indicates that the development of fibrosis depends on the pattern of macrophage polarization and the persistence of inflammatory stimuli, as illustrated in Figure 7. Although it was technically challenging to precisely quantify infiltrating CD80^+^ or CD206^+^ macrophages in skin sections, the immunohistochemical data on total and M1/M2 macrophages, together with the marked reduction of these subsets by darapladib, are noteworthy (Figure 8). We further confirmed that darapladib directly modulates M1/M2 polarization in BMDMs cultures, as demonstrated by FACS analysis.

## 5. Conclusions

Our data demonstrate that darapladib exerts anti-fibrotic effects in the skin by inhibiting LPC production, reducing ROS generation, inhibiting collagen deposition, and reversing myofibroblast activation toward a more fibroblast-like phenotype. Darapladib attenuated the early inflammatory response by inhibiting M1 macrophage activation and suppressed sustained M2 macrophage hyperactivation at later stages of injury. Therefore, darapladib may represent a promising therapeutic option for patients with post-radiation dermatitis and skin fibrosis.

## 6. Patents

A patent for the results has been registered at the Ministry of Intellectual Property in Republic of Korea (10-2891833).

## Figures and Tables

**Table 1:** List of antibodies used in the experiments.

Antibody	Isotype	Clone	Source	Catalog No.
CD11b	Rat IgG2b	m1/70	BD Biosciences	#553312
Cy6C	Rat IgG2c	HK1.4	Biolegend	#128027
CD45	Rat IgG2b	30-F11	Biolegend	#103133
CD80	Armenian Hamster IgG	16-10A1	Biolegend	#104713
CD206	Rat IgG2a	C068C2	Biolegend	#141719

## Data Availability

All data generated or analyzed during this study are included in this published article.

## Abbreviations

The following abbreviations are used in this manuscript:

Lp-PLA2	lipoprotein-associated phospholipase A2
RISI	radiation-induced skin injury
IR	ionizing radiation
EndoMT	endothelial-to-mesenchymal transition
FMT	fibroblast-to-myofibroblast transition
LPC	lysophosphatidylcholine
ECM	extracellular matrix
TNF-α	tumor necrosis factor-alpha
IL-1β	interleukin-1beta
TGF-β	transforming growth factor-beta
α-SMA	alpha-smooth muscle actin
iNOS	inducible nitric oxide synthase
TEWL	transepidermal water loss
DWAT	dermal white adipose tissue
